# Spraying KH_2_PO_4_ Alleviates the Damage of Spring Low-Temperature Stress by Improving the Physiological Characteristics of Wheat Flag Leaves

**DOI:** 10.3390/ijms252111542

**Published:** 2024-10-27

**Authors:** Xiang Chen, Ying Weng, Tiantian Chen, Wenci Dai, Zhiwei Tang, Hongmei Cai, Baoqiang Zheng, Jincai Li

**Affiliations:** 1 College of Agronomy, Anhui Agricultural University, Hefei 230036, China; cx2468@ahau.edu.cn (X.C.); wengying0918@stu.ahau.edu.cn (Y.W.); tian18305565649@163.com (T.C.); 15637625302@stu.ahau.edu.cn (W.D.); 22720552@stu.ahau.edu.cn (Z.T.); chm2023@ahau.edu.cn (H.C.); zhengbaoqiang@ahau.edu.cn (B.Z.); 2 Jiangsu Collaborative Innovation Centre for Modern Crop Production, Nanjing 210095, China

**Keywords:** potassium dihydrogen phosphate, *Triticum aestivum* L., cold stress, anatomical structure, photosynthetic performance, yield

## Abstract

The low-temperature stress (LTS) in spring results in tremendous yield loss in wheat production, and the application of potassium dihydrogen phosphate (KH_2_PO_4_) can alleviate stress-induced damage. However, the underlying effect of spraying KH_2_PO_4_ on the physiological characteristics of wheat flag leaves under spring LTS remains unclear. In this study, we investigated the effect of spraying KH_2_PO_4_ on flag leaf physiological traits and yield under spring LTS, including treatments at 15 °C and spraying H_2_O (CK), treatment at −4 °C and spraying H_2_O (LT1), and treatment at −4 °C and spraying KH_2_PO_4_ (LT2). The results showed that spraying KH_2_PO_4_ significantly increased the activities of the superoxide dismutase (SOD), the peroxidase (POD), and the catalase (CAT), and reduced malondialdehyde (MDA) content in the flag leaves. Compared to LT1, the SOD, POD, and CAT activities in the flag leaves of the Yangnong19 (YN19) and Xinmai26 (XM26) via LT2 increased by 5.5%, 10.9%, and 3.9%, and 5.4%, 9.2%, and 4.4%, respectively, and the MDA content of the YN19 and XM26 decreased by 10.5% and 9.1%, respectively, at 0–12 d after low temperature treatment (DALTT). Spraying KH_2_PO_4_ appreciably alleviated damage to the leaf cell morphology and tissue integrity, and increased the accumulation of proline and soluble protein, the chlorophyll content, and the activities of Ribulose–1,5–bisphosphate carboxylase and phosphoenolpyruvate carboxykinase. The net photosynthetic rate in the flag leaves of the YN19 and XM26 via LT2 increased by 37.9% and 35.9%, respectively, at 0–12 DALTT, compared to LT1. Moreover, spraying KH_2_PO_4_ reduced the yield loss rate of the YN19 and XM26 by 13.06% and 16.72%, respectively. The present study demonstrates that spraying KH_2_PO_4_ can enhance wheat resistance to spring LTS and maintain the photosynthetic capacity of flag leaves, alleviating the negative effects of LTS on grain yield.

## 1. Introduction

Wheat (*Triticum aestivum* L.) is one of the most widely cultivated cereal crops in the world, providing a nutritious and staple food for approximately 40% of the global population, and the stability of wheat production is crucial to ensure food security and reduce starvation [1,2]. Low-temperature stress (LTS) is an important limiting factor affecting wheat growth and development in wheat-growing regions worldwide [3,4,5,6,7]. Global warming is a huge challenge to wheat production due to the increasing frequency and intensity of LTS, especially spring LTS in the Huang-Huai-Hai plain, the largest wheat production area in China [3,8].

Many studies have shown that LTS hampers wheat growth, development, and yield formation by altering their inherent physiological and biochemical processes and pathways [8,9]. Photosynthesis is the source of crop metabolism and energy transformation, and is also one of the most sensitive physiological and biochemical processes to LTS [10]. Hence, photosynthesis-related parameters have been used to detect and quantify the damage caused by LTS in wheat. Spring LTS damages the cell morphology, and chloroplast and mitochondrial structures of leaves and affects the integrity of the cell structure [11]. Most recently, Zhang et al. [12] pointed out that the chlorophyll a, chlorophyll b, total chlorophyll, and carotenoids contents of wheat leaves showed a significant decline due to the increased severity of spring LTS (0 °C to −3 °C). LTS also decreased the enzymatic activities (i.e., 1,5-diphosphate ribulose carboxylase, phosphoenolpyruvate carboxylase) associated with photosynthetic carbon assimilation, inhibiting the Calvin cycle, thus reducing the photosynthetic rate [13]. Li et al. [14] found that spring LTS significantly decreased gas exchange rates and the maximum quantum efficiency of photosystem II in wheat leaves, resulting in a 5 to 14% in grain yield loss. This indicates that LTS mainly reduces the photosynthetic rate of wheat leaves by altering the structure of chloroplasts, inhibiting the key enzymes involved in photosynthetic carbon assimilation, ultimately leading to yield losses in wheat. Thus, it is urgent to create effective prevention and defensive measures to maintain sustainable wheat production.

Diverse strategies are able to effectively enhance the resistance to spring LTS in wheat [15,16,17,18,19]. The exogenous application of chemical compounds such as salicylic acid [20], abscisic acid [21], trehalose [22], 6-benzylamino adenine [23], melatonin [24], and potassium dihydrogen phosphate (KH_2_PO_4_) [25,26] can also greatly improve wheat resistance to LTS. Notably, KH_2_PO_4_, as a phosphate and potassium complex, is widely used as a foliar fertilizer to replenish phosphate and potassium in plants and improve the wheat’s resistance to temperature stress in wheat production [27]. Foliar spraying KH_2_PO_4_ after anthesis can enhance the heat stress resistance of wheat by improving the photosynthetic capacity of functional leaves, promoting dry matter production and alleviating grain yield loss [28]. Most recently, Huang et al. [25] reported that KH_2_PO_4_ application can alleviate the damage from spring LTS, as it can enhance the relative chlorophyll contents of wheat flag leaves, root activity, and antioxidant enzyme activities, and reduce malondialdehyde (MDA) content in both flag leaves and roots during post-anthesis, thus delaying the aging processes of flag leaves and roots. Dai et al. [26] found that spraying KH_2_PO_4_ after spring LTS can increase the fertile grain number and grain weight of the base and top spikelets with inferior grain positions to reduce the yield loss. Su et al. [29] further indicated that KH_2_PO_4_ application can alleviate the damage of spring LTS to young spikes and improve the seed setting rate. Based on previous studies, spraying KH_2_PO_4_ has been verified to be an effective means to deal with spring LTS in wheat. Even so, research about the effects of KH_2_PO_4_ on the anatomical structure and physiological characteristics of functional leaves in wheat under spring LTS was limited.

In this present study, we conducted a controlled phytotron experiment using two wheat cultivars with contrasting spring LTS resistance and foliar KH_2_PO_4_ after spring LTS. The study testified the following hypotheses: (1) spraying KH_2_PO_4_ can enhance reactive oxygen species metabolism and osmoregulation substance contents in wheat flag leaves under spring LTS; (2) spraying KH_2_PO_4_ can alleviate damage to the cell morphology and tissue integrity and maintain the photosynthetic capacity of wheat flag leaves after spring LTS.

## 2. Results

### 2.1. Antioxidant Enzyme Activity

As the plant grows, the activities of the superoxide dismutase (SOD), peroxidase (POD), and catalase (CAT) in the flag leaves of the two wheat varieties, YN19 and XM26, displayed patterns of initially increasing and then declining, while the malondialdehyde (MDA) content showed a continuous upward trend (Table 1). Compared to CK, the activities of SOD, POD, and CAT in the YN19 and XM26 flag leaves in 0–12 DALTT, increased by 50.7%, 72.9%, and 15.6%, and 49.0%, 64.1%, and 20.1%, respectively, via LT1, and increased by 58.2%, 88.9%, and 22.0%, and 56.5%, 77.5%, and 25.4%, respectively, via LT2. Similarly, the MDA content of the flag leaves of the YN19 and XM26 increased by 69.7% and 78.1%, respectively, via LT1, and increased by 51.0% and 61.3%, respectively, via LT2, compared to CK. Compared to LT1, the activities of SOD, POD, and CAT in the YN19 and XM26 flag leaves in 0–12 DALTT, increased by 5.5%, 10.9%, and 3.9%, and 5.4%, 9.2%, and 4.4%, respectively, via LT2, whereas the MDA content in the flag leaves of the YN19 and XM26 decreased by 10.5% and 9.1% via LT2.

### 2.2. Osmotic Adjustment Substances

The proline (Pro) and soluble protein (SP) contents in the flag leaves of two wheat varieties exhibited a continuous increase as the growth stages advanced (Table 2). At 0–12 DALTT, the Pro and SP contents increased by 9.1% and 49.2%, and 13.0% and 46.0%, respectively, via LT1, and increased by 15.3% and 66.7% and 16.9% and 62.6%, respectively, via LT2, compared to CK, while the Pro and SP contents of the YN19 and XM26 via LT2 increased by 5.7% and 12.6%, and 3.4% and 12.2%, respectively, compared to LT1.

### 2.3. Flag Leaf Anatomy

**Figure 1:**
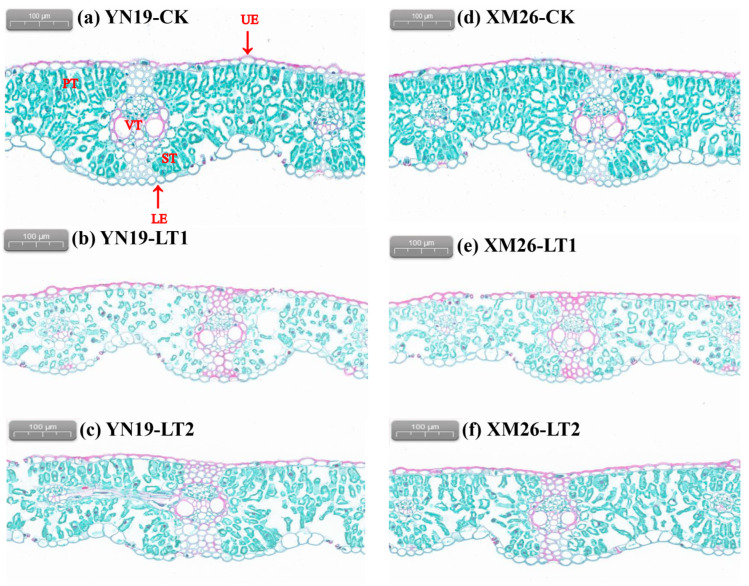
Comparison of flag leaf anatomy between Yannong19 (YN19) and Xinmai26 (XM26) in CK (**a**,**d**), LT1 (**b**,**e**), and LT2 (**c**,**f**). Legend: UE—upper epidermis; LE—lower epidermis; PT—palisade tissue; ST—spongy tissue; VT—vascular tissue. CK refers to treatment at 15 °C and spraying H_2_O; LT1 refers to treatment at −4 °C and spraying H_2_O; LT2 refers to treatment at −4 °C and spraying KH_2_PO_4_.

Following the application of safranin O-fast green staining solution to wheat flag leaves, the cell walls in the upper epidermis and the vascular tissues appeared red, due to lignification, while in the palisade tissues, the spongy tissues, and the lower epidermis, the cell walls with cellulose appeared green. The mesophyll cells of the flag leaves in CK exhibited intact structure, differentiated into two parts: palisade tissue and spongy tissue (Figure 1). The long cylindrical cells, closely arranged near the upper epidermis, formed the palisade tissue, while the irregularly shaped cells, loosely arranged near the lower epidermis, constituted the spongy tissue. In LT1, the mesophyll cell structure was damaged; the intercellular spaces were enlarged, and the number of both palisade and spongy tissue cells was reduced and shortened, whereas LT2 alleviated the damage caused by spring LTS on the mesophyll cell structure, showing smaller intercellular spaces compared to LT1, with an increased number and length of both palisade and spongy tissue cells.

The spring LTS significantly reduced the leaf thickness, upper epidermis thickness, lower epidermis thickness, palisade tissue thickness, spongy tissue thickness, leaf structure compactness, and leaf structure density in the flag leaves of two wheat varieties, while spraying KH_2_PO_4_ significantly increased the aforementioned mesophyll cell structures (Table 3). Compared to CK, the leaf thickness, upper epidermis thickness, lower epidermis thickness, palisade tissue thickness, spongy tissue thickness, tightness of the blade structure, and blade structure dredging density of the YN19 and XM26 via LT1 significantly decreased by 18.0%, 53.4%, 44.4%, 46.0%, 42.5%, 34.5%, and 30.4%, and 18.1%, 51.9%, 40.2%, 46.7%, 36.6%, 33.3%, and 23.8%, respectively. Compared to LT1, the leaf thickness, upper epidermis thickness, lower epidermis thickness, palisade tissue thickness, spongy tissue thickness, tightness of the blade structure, and blade structure dredging density of the YN19 and XM26 via LT2 significantly increased by 10.4%, 49.3%, 41.1%, 35.7%, 30.8%, 26.3%, and 18.8%, and 11.9%, 47.0%, 40.8%, 35.3%, 27.7%, 22.2%, and 12.5%, respectively. 

### 2.4. Ultrastructure of Chloroplasts

**Figure 2:**
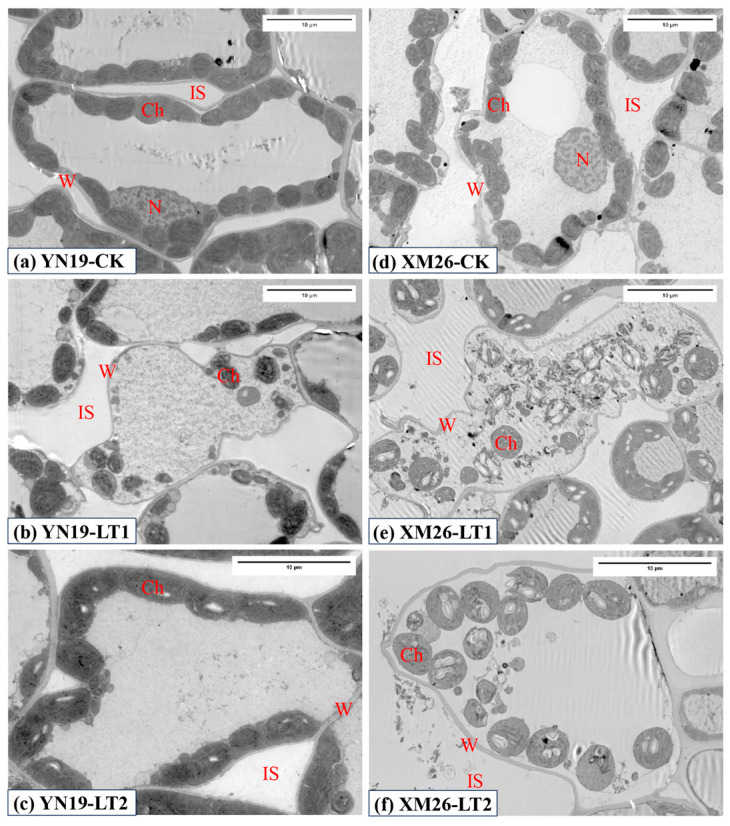
Comparison of ultrastructure of flag leaf mesophyll cell between Yannong19 (YN19) and Xinmai26 (XM26) in CK (**a**,**d**), LT1 (**b**,**e**) and LT2 (**c**,**f**). Legend: Ch—chloroplast; W—cell wall; N—nucleus; IS—intercellular space. CK refers to treatment at 15 °C and spraying H_2_O; LT1 refers to treatment at −4 °C and spraying H_2_O; LT2 refers to treatment at −4 °C and spraying KH_2_PO_4_.

As can be seen from Figure 2, the flag leaf flesh cells of the two wheat varieties had complete structures, and the chloroplasts were neatly arranged around the cell wall, with a clear oval structure in CK. Under LT1, the mesophyll cell walls became deformed, the number of chloroplasts decreased, some chloroplasts disintegrated, and their contents leaked out. Under LT2, the number of chloroplasts increased compared to LT1; they were slightly swollen and no longer neatly arranged around the cell wall, with the cell wall being slightly deformed. The spring LTS significantly reduced the number of chloroplasts, the chloroplast area, and the chloroplast length/width (Table 4). Spraying KH_2_PO_4_ significantly increased the number of chloroplasts, the chloroplast area, and the chloroplast length/width in the flag leaves of the two wheat varieties under spring LTS. Compared to CK, the number of chloroplasts, the chloroplast area, and the chloroplast length/width of the YN19 and XM26 via LT1 significantly decreased by 64.4%, 52.7%, and 31.8%, and 62.8%, 54.7%, and 33.5%, respectively. Compared to LT1, the number of chloroplasts, the chloroplast area, and the chloroplast length/width of the YN19 and XM26 via LT2 significantly increased by 95.3%, 39.9%, and 15.8%, and 94.8%, 44.8%, and 21.9%, respectively.

**Figure 3:**
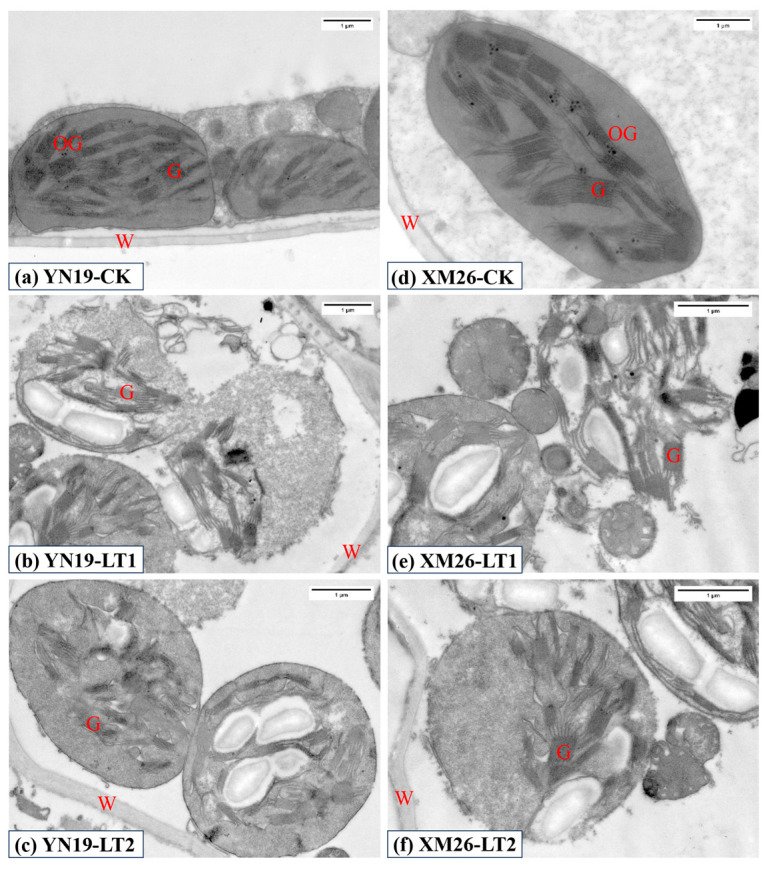
Comparison of ultrastructure of flag leaf meat cells between Yannong19 (YN19) and Xinmai26 (XM26) in CK (**a**,**d**), LT1 (**b**,**e**) and LT2 (**c**,**f**). Legend: W—cell wall; G—grana; OG—osmiophilic granule. CK refers to treatment at 15 °C and spraying H_2_O; LT1 refers to treatment at −4 °C and spraying H_2_O; LT2 refers to treatment at −4 °C and spraying KH_2_PO_4_.

As shown in Figure 3, the chloroplast grana of the flag leaves of the two wheat varieties under CK were arranged neatly, with tight grana lamella, and neatly stacked. Under LT1, the chloroplasts were dilated and damaged, and the grana lamella was scattered and disintegrated. Under LT2, the grana lamella was compact and did not disintegrate, and the chloroplasts were slightly expanded. The spring LTS significantly reduced the number of grana, the number of grana lamellae, and the grana thickness in the chloroplasts of the flag leaves (Table 5). Spraying KH_2_PO_4_ significantly increased the number of grana and grana lamellae, and the grana thickness in the chloroplasts of the flag leaves under spring LTS. Compared to CK, the number of grana and grana lamellae, and the grana thickness of the YN19 and XM26 via LT1 significantly decreased by 56.9%, 59.7%, and 44.0%, and 69.0%, 67.9%, and 38.1%, respectively. Compared to LT1, the number of grana and grana lamellae, and the grana thickness in the flag leaves of the YN19 and XM26 via LT2 significantly increased by 64.3%, 88.1%, and 35.7%, and 111.2%, 138.8%, and 30.8%, respectively. 

### 2.5. Photosynthetic Pigment and Photosynthetic Enzyme Activity

**Figure 4:**
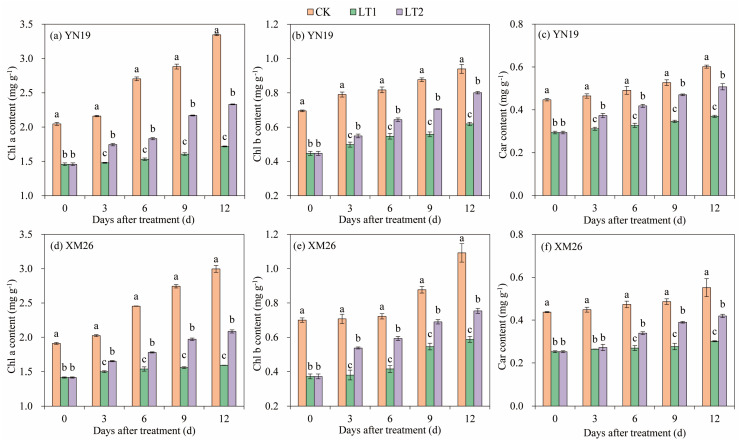
Comparison of Chl a (**a**,**d**), Chl b (**b**,**e**) and Car (**c**,**f**) contents of flag leaves between Yannong19 (YN19) and Xinmai26 (XM26) in different treatments. The standard deviation (SD) values from the three replicates are represented by error bars in the figures, and different lowercase letters indicate significant differences at the *p <* 0.05 level within each day of treatment. Chl a—chlorophyll a; Chl b—chlorophyll b; Car—carotenoid. CK refers to treatment at 15 °C and spraying H_2_O; LT1 refers to treatment at −4 °C and spraying H_2_O; LT2 refers to treatment at −4 °C and spraying KH_2_PO_4_.

The chlorophyll a (Chl a), chlorophyll b (Chl b), and carotenoid (Car) contents in the flag leaves of the two wheat varieties exhibited a rising trend as the growth stages progressed (Figure 4). Compared to CK, the contents of Chl a, Chl b, and Car in the YN19 and XM26 flag leaves at 0–12 DALTT decreased by 39.3%, 35.2%, and 34.7% and 35.9%, 43.9%, and 43.0%, respectively, via LT1, and increased by 27.1%, 24.3%, and 19.0%, and 26.1%, 28.2%, and 30.8%, respectively, via LT2. Compared to LT1, the contents of Chl a, Chl b, and Car in the YN19 and XM26 flag leaves at 0–12 DALTT increased by 21.7%, 16.7%, and 24.2%, and 16.7%, 27.8%, and 21.8%, respectively, via LT2.

**Figure 5:**
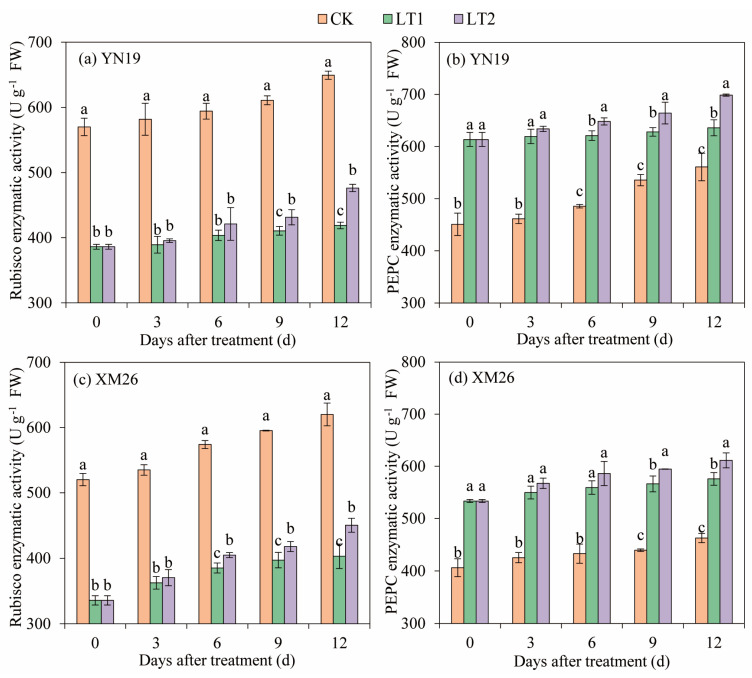
Comparison of activities of Rubisco (**a**,**c**) and PEPC (**b**,**d**) of flag leaves between Yannong19 (YN19) and Xinmai26 (XM26) in different treatments. The standard deviation (SD) values from three replicates are represented by error bars in figures, and different lowercase letters indicate significant differences at the *p <* 0.05 level within each day of treatment. Rubisco—Ribulose–1,5–bisphosphate carboxylase; PEPC—phosphoenolpyruvate carboxykinase. CK refers to treatment at 15 °C and spraying H_2_O; LT1 refers to treatment at −4 °C and spraying H_2_O; LT2 refers to treatment at −4 °C and spraying KH_2_PO_4_.

The activities of Ribulose–1,5–bisphosphate carboxylase (Rubisco) and phosphoenolpyruvate carboxykinase (PEPC) in the flag leaves of the two wheat varieties showed consistent upward trends as the growth stages advanced (Figure 5). At 0–12 DALTT, the Rubisco activities of the YN19 and XM26 flag leaves via LT1 and LT2 were lower than that in CK, decreased by 33.1% and 29.9% and 33.8% and 30.6%, respectively, whereas the activities of the PEPC via LT1 and LT2 were higher than that in CK, increased by 25.8% and 31.1% and 28.6% and 33.5%, respectively. At 0–12 DALTT, the Rubisco activity in the flag leaves of the YN19 and XM26 via LT2 showed an increasing trend compared to LT1, rising by 4.9% and 4.8%, respectively, while the PEPC activity in LT2 increased by 4.5% and 3.8%, respectively, compared to LT1.

### 2.6. Photosynthetic Parameters

**Figure 6:**
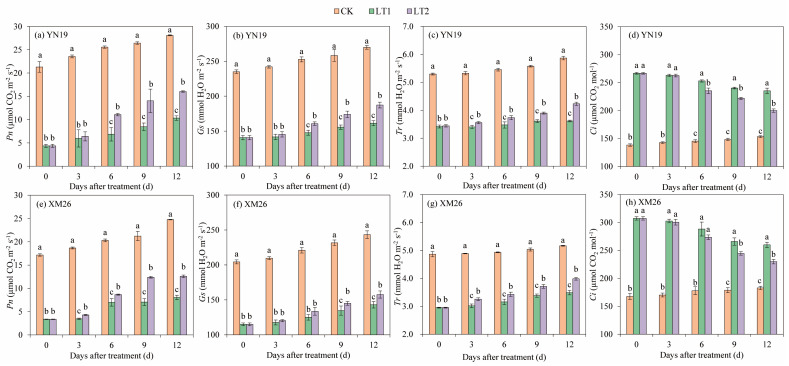
Comparison of Pn (**a**,**e**), Gs (**b**,**f**), Tr (**c**,**g**), and Ci (**d**,**h**) of flag leaves between Yannong19 (YN19) and Xinmai26 (XM26) in different treatments. The standard deviation (SD) values from three replicates are represented by error bars in figures, and different lowercase letters indicate significant differences at the *p <* 0.05 level within each day of treatment. Pn—net photosynthetic rate; Gs—stomatal conductivity; Tr—transpiration rate; Ci—intercellular CO_2_ concentration. CK refers to treatment at 15 °C and spraying H_2_O; LT1 refers to treatment at −4 °C and spraying H_2_O; LT2 refers to treatment at −4 °C and spraying KH_2_PO_4_.

As the growth process advanced, the net photosynthetic rate (Pn), the stomatal conductivity (Gs), and the transpiration rate (Tr) of the flag leaves in the two wheat varieties tended to increase overall, while intercellular CO_2_ concentration (Ci) followed a gradual decreasing trend (Figure 6). At 0–12 DALTT, compared to CK, the Pn, Gs, and Tr in the flag leaves of the YN19 and XM26 significantly decreased by 71.7%, 40.6%, and 36.3%, and 72.3%, 42.8%, and 35.7%, respectively, via LT1, and by 59.8%, 36.0%, and 31.5% and 61.1%, 39.7%, and 30.5%, respectively, via LT2. Compared to CK, the Ci in the YN19 and XM26 flag leaves increased by 73.3% and 63.8%, and 62.7% and 55.2% via LT1 and LT2, respectively. The Pn, Gs, and Tr in the flag leaves of the YN19 and XM26 rose via LT2 by 37.9%, 7.9%, and 7.5%, and 35.9%, 5.4%, and 8.1%, respectively, while the Ci decreased by 6.0% and 5.1%, respectively, via LT2 compared to LT1. 

### 2.7. Yield and Its Component Factors

The spring LTS significantly reduced the grain number per spike (GNPS), 1000-grain weight (TGW), and yield of two wheat varieties, while the KH_2_PO_4_ application increased these values (Table 6). Compared to CK, the GNPS, the TGW, and the grain yield per stem (GYPS) of the YN19 and XM26 were significantly reduced by 33.8%, 25.0%, and 36.4% and 60.4%, 29.9%, and 52.3%, respectively, via LT1, and by 18.0%, 9.6%, and 23.6% and 43.8%, 17.6%, and 35.6%, respectively, via LT2. Compared to LT1, the GNPS, TGW, and the GYPS of the YN19 and XM26 via LT2 significantly increased by 23.9%, 20.4%, and 20.2% and 42.1%, 17.6%, and 35.0%, respectively. The yield loss rates of the YN19 and XM26 via LT1 and LT2 were 36.6% and 23.5%, and 52.4% and 35.6%, respectively. The yield recovery rates in the YN19 and XM26 were 35.7% and 31.9%, respectively.

### 2.8. Correlation Analysis

**Figure 7:**
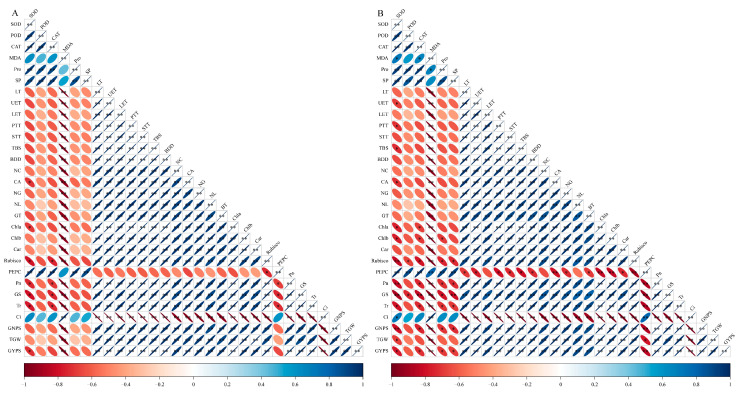
Correlation analysis of flag leaf indexes of Yannong19 (YN19, **A**) and Xinmai26 (XM26, **B**). *, **mean *p* < 0.05, *p* <0.01. Red indicates negative correlation and blue indicates positive correlation. The upper left and lower right direction of the ellipse represents negative correlation, the upper right and lower left direction represents positive correlation, and the smaller the ellipse, the more significance it is. Legend: LT—leaf thickness; UET—upper epidermis thickness; LET—lower epidermis thickness; PTT—palisade tissue thickness; STT—spongy tissue thickness; TBS—tightness of the blade structure; BDD—blade structure dredging density; NC—number of chloroplasts; CA—chloroplast area; NG—number of grana; NL—number of grana lamellae; GT—grana thickness; GNPS—Grain number per spike; TGW—1000-grain weight; GYPS—grain yield per stem; YLR—yield loss rate; YRR—yield recovery rate.

A correlation analysis was conducted on the antioxidant enzyme activity, the osmotic adjustment substances, the anatomical structure, the photosynthetic pigments, the photosynthetic enzymes, the photosynthetic parameters in the flag leaves of the YN19 at 12 DALTT, and the yield (Figure 7A). The antioxidant enzyme activity (SOD, POD, CAT) in the flag leaves of the YN19 showed a negative correlation with the yield (GNPS, TGW, GYPS), and the MDA displayed a highly significant and negative correlation with the yield. The osmotic adjustment substances (Pro, SP) were negatively correlated with the yield, while the ultrastructure and anatomy exhibited highly significant and positive correlations with the yield. The photosynthetic pigments and Rubisco activity were also positively correlated with the yield, whereas the PEPC activity showed a negative correlation with the yield. The photosynthetic parameters (Pn, Gs, Tr) were highly significantly and positively correlated with the yield, while the Ci exhibited a highly significant and negative correlation with the yield.

Similarly, a correlation analysis was conducted on the flag leaves of the XM26 (Figure 7B). The SOD showed a significant and negative correlation with the yield; the POD was negatively correlated, and the CAT exhibited a significant and negative correlation with the yield (GNP, GYPS) and a negative correlation with the TGW. The MDA showed a highly significant and negative correlation with the yield. The osmotic adjustment substances (Pro, SP) had significant negative correlations with the yield (GNP, GYPS) and negative correlations with the TGW. The ultrastructure and anatomy were highly significantly and positively correlated with the yield, and the photosynthetic pigments and Rubisco were also highly positively correlated with the yield, while the PEPC showed significant and negative correlations. The photosynthetic parameters (Pn, Gs, Tr) were highly significantly and positively correlated with the yield, whereas the Ci was highly negatively correlated with the yield. Therefore, the anatomical structure of the flag leaf and photosynthetic physiological indexes of the wheat were closely related to the yield.

## 3. Discussion

### 3.1. Effects of Spraying KH_2_PO_4_ on Antioxidant Enzyme Activity and Osmotic Adjustment Substances of Wheat Flag Leaves Under Spring LTS

LTS induces a large amount of reactive oxygen species (ROS) in plant cells. Excessive ROS accumulation reacts with lipids, resulting in the formation of MDA, a lipid peroxidation product that indirectly reflects the extent of membrane lipid peroxidation [30,31]. The present study also found that spring LTS significantly increased the MDA content in wheat flag leaves. Su et al. [29] demonstrated that KH_2_PO_4_ application before LTS at the booting stage significantly increased antioxidant enzyme activities in wheat leaves, reduced the MDA content, and enhanced the leaf resistance, mitigating damage to the membrane lipids caused by LTS. Similarly, Huang et al. [25] reported that KH_2_PO_4_ application after LTS at the booting stage significantly increased antioxidant enzyme activities in post-anthesis flag leaves and reduced the MDA content. Our results were consistent with previous studies; spraying KH_2_PO_4_ increased the activities of SOD, POD, and CAT, and reduced the MDA content. This may be due to the fact that KH_2_PO_4_ application improves phosphorus and potassium metabolism in plants, and further increases antioxidant enzyme activities, effectively eliminating ROS generated under LTS and reducing membrane lipid peroxidation [29,32,33]. 

Pro accumulation is an essential metabolic adaptation mechanism of plants under abiotic stress [34]. Li et al. [35] reported that LTS increased the levels of Pro and SP in wheat functional leaves, in agreement with the present study. Xu et al. [17] demonstrated that optimizing phosphorus application could enhance the activities of carbon and nitrogen metabolism enzymes in young wheat spikes, increase the accumulation of SP and Pro, and mitigate the damage to spike development caused by LTS. Our study also showed that spraying KH_2_PO_4_ further enhanced the Pro and SP levels under spring LTS. This might be attributed to the increased osmotic adjustment substances triggering the formation of abscisic acid, inducing protein synthesis, and boosting the stress resistance in wheat plants [36], but further research on the hormone metabolism mechanism is required.

### 3.2. Effects of Spraying KH_2_PO_4_ on Anatomical Structure of Wheat Flag Leaves Under Spring LTS

The leaf is the most susceptible organ in plants to external environment changes, and its anatomical structure can reflect the plant’s response to variations in environmental conditions. The arrangement of wheat leaf mesophyll cells can become irregular under LTS [37]. The present study indicates that spring LTS decreased the number and thickness of palisade tissue cells and spongy tissue cells, resulting in the damage of the meat cell structure, the enlargement of the cell space, and the destruction of the integrity of the leaf cell structure. Sun et al. [38] reported that plant leaves have thicker palisade tissue and more chlorophyll under phosphorus fertilizer application, enhancing the plant’s ability to assimilate carbon. This study also found that spraying KH_2_PO_4_ increased the thickness of the palisade and spongy tissues, alleviating the damage to mesophyll cells caused by spring LTS. It is possible that spraying KH_2_PO_4_ replenishes wheat with phosphorus and potassium nutrition, and promotes the formation of thickened angular tissue, and enhances the stability of leaf structure and stress resistance [16,17,39].

The ultrastructure of mesophyll cells determines the photosynthetic capacity in plants, with chloroplasts serving as the primary site of photosynthesis. The tight and orderly arrangement of thylakoid membranes within chloroplasts enhances the photosynthetic rate of leaves [40]. Liu et al. [41] revealed that cold stress significantly reduced the area of wheat chloroplasts, the number of thylakoid granum, and the thickness of the granum. The present study found that spring LTS deformed the cell walls of flag leaf mesophyll cells and significantly reduced the number of chloroplasts, the thylakoid granum, the thylakoid layers, and the granum thickness. The thylakoid layers became disordered, accompanied by signs of degradation, while KH_2_PO_4_ application alleviated the damage to the chloroplast structure caused by LTS. Wang et al. [42] showed that increasing phosphorus levels in phosphorus-inefficient soybean genotypes enlarged chloroplasts and significantly increased the number of thylakoid layers, with the denser stacking of thylakoid membranes. Therefore, spraying KH_2_PO_4_ may boost the number of chloroplasts and thylakoid layers, leading to a more tightly arranged thylakoid structure, improving photosynthetic efficiency and enhancing the plant’s stress resistance [42,43].

### 3.3. Effect of Spraying KH_2_PO_4_ on Photosynthetic Characteristics of Wheat Flag Leaves Under Spring LTS

Chlorophyll and carotenoids play important roles in the photosynthetic tissues of plants [44]. Zhang et al. [12] reported that LTS led to a decreased photosynthetic pigment contents in wheat leaves. In the present study, the Chl a, Chl b, and Car contents of wheat flag leaves also decreased under spring LTS compared to CK. This may be attributed to the reduced photosynthetic pigment synthesis enzyme activities, leading to a decrease in pigment contents [45]. The present study further analyzed the response of Rubisco and PEPC to spring LTS. Rubisco and PEPC are important CO_2_-fixing enzymes in the Calvin cycle of photosynthesis in C3 plants and play important roles in the biochemical photosynthesis in wheat under adverse stress [13,46]. Van Kiet and Nose [47] reported that low temperatures could inhibit the activity of Rubisco in rice, resulting in a lowered photosynthetic capacity. Similar results also were observed in this experiment; spring LTS reduced Rubisco activity in the XM26 and YN19. On the contrary, the PEPC activity was increased under spring LTS, which is consistent with Fan et al. [13]. Additionally, in the current study, we observed that spraying KH_2_PO_4_ was beneficial to maintaining high levels of photosynthetic pigments and photosynthetic enzyme activities under spring LTS. The chlorophyll contents, Rubisco and PEPC activities of the flag leaves of the YN19 and XM26 via LT2 were higher than those via LT1. This may be due to the crucial role of phosphorus in the formation and functioning of photosynthetic organs, as it is involved in nearly all photosynthetic processes, which could improve the chlorophyll contents and the activities’ photosynthesis-related enzymes in wheat leaves under spring LTS [48,49].

Photosynthetic parameters, particularly net photosynthetic rate, could intuitively represent the level of photosynthetic performance in plant leaves. Li et al. [14] found that the Pn of wheat leaves was significantly lower under LTS compared to the ambient temperature. Zhang et al. [50] reported that spring LTS reduced the Pn of wheat leaves, and the Pn gradually decreased with the extension of the treatment time and the decreased temperature, in agreement with the present study. Our study showed that spraying KH_2_PO_4_ could contribute to increasing trends in the Pn in flag leaves under spring LTS. As the essential elements for wheat, phosphorus, and potassium play crucial roles in various metabolic pathways, such as in photosynthesis and stomatal movement regulation [16,51]. Spraying KH_2_PO_4_ may replenish wheat plants with phosphate and potassium, and improve the photosynthetic physiological and metabolic processes, leading to increased Pn in flag leaves under spring LTS [43]. Furthermore, we found that the Gs of wheat flag leaves increased with spraying KH_2_PO_4_ under spring LTS, probably due to the increased proportion of the stomatal opening of the leaves [27].

### 3.4. Effect of Spraying KH_2_PO_4_ on Wheat Yield Under Spring LTS

Many studies have demonstrated that spring LTS can depress the development of spikelets and aggravate the abortion of florets, and severe spring LTS can result in wheat yield loss of over 50% [11,50,52]. Our study showed that spring LTS significantly decreased the GNPS, TGW, and GYPS in two varieties. In addition, the reduction in the grain yield of the YN19 was lower than that in the XM26, suggesting that YN19 has a stronger self-protection mechanism and is more tolerant to spring LTS than XM26 [11]. Under spring LTS conditions, the GNPS and TGW, as well as the GYPS were increased by spraying KH_2_PO_4_ (Table 6). This may be attributed to foliar spraying KH_2_PO_4_ not only increasing the activities of antioxidant enzymes and decreasing the MDA content in wheat flag leaves (Table 1), but also causing the accumulation of Pro and soluble protein (Table 2). This contributed to improving reactive oxygen metabolism and osmotic regulation ability, and maintaining the cell morphology and tissue integrity of wheat flag leaves (Figure 1, Figure 2 and Figure 3). The elevation in leaf photosynthetic pigment content and photosynthetic enzyme activity facilitated photosynthetic recovery, ultimately alleviating the adverse effects of spring LTS on the wheat yield. This is consistent with previous findings that spraying KH_2_PO_4_ could effectively increase the wheat grain yield in the field under natural spring LTS [26]. However, the present study only focuses on the effects of spraying KH_2_PO_4_ on the physiological characteristics of wheat flag leaves; further studies are needed to explore the physiological effect of spraying KH_2_PO_4_ on improving spikelet development and setting.

## 4. Materials and Methods

### 4.1. Plant Materials and Treatment

Following a previous study [11], this experiment utilized Yannong19 (YN19, strongly resistant to spring LTS) and Xinmai26 (XM26, susceptible to spring LTS) as the test materials. The YN19 and XM26 were bred by Yantai Academy of Agricultural Sciences and Xinxiang Academy of Agricultural Sciences of Henan Province, respectively [50]. The experiment was carried out from November 2022 to May 2023 at the High-Tech Agricultural Park of Anhui Agricultural University (31°55′38″ N, 117°14′44″ E). Potting soil was taken from 0 to 20 cm topsoil in the field. The soil type was brown loam, with a pH value of 5.7, and the contents of organic matter, total nitrogen, available phosphorus and available potassium were 14.53 g/kg, 1.03 g/kg, 18.25 mg/kg, and 256.02 mg/kg, respectively. A field pot experiment was designed, with soil that was sieved and placed into PVC pots (diameter of 26 cm, height of 35 cm, 5 drainage holes). According to Jiang et al. [53] and Lin et al. [54], each pot was filled with 8 kg of air-dried soil mixed with 4.32 g of compound fertilizer (N: P: K = 15:15:15). The sowing date was 4 November 2022, and the sowing depth was 3 cm. Each pot was evenly sown with 18 vital and uniform wheat seeds, then covered with an additional 2 kg of soil. Surrounding each pot, wheat of the same variety was planted to maintain consistent environmental conditions. At the three-leaf stage (9 December 2022), seedlings were thinned to 9 uniformly growing wheat plants per pot. According to Dai et al. [26], urea (0.87 g/pot) was applied at the jointing stage to support further growth.

**Figure 8:**
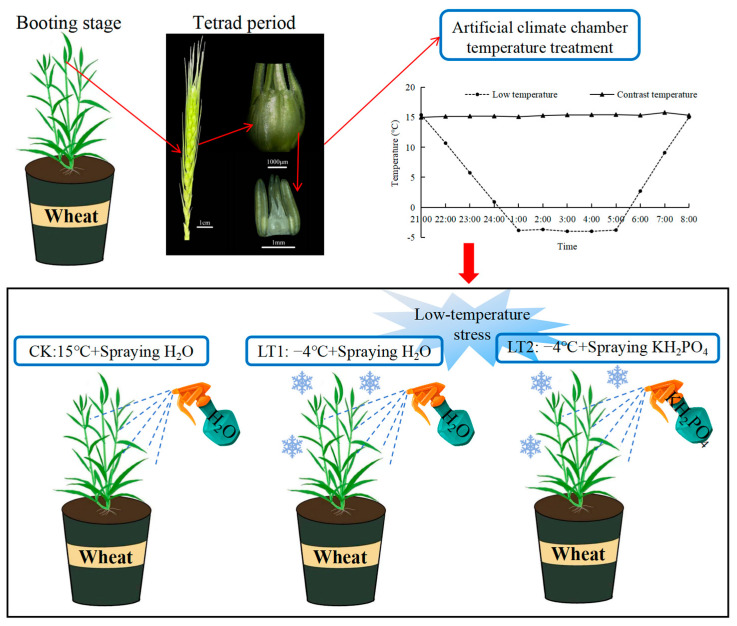
A schematic diagram outlining the experimental design and treatments.

The experiment was designed with two varieties, three treatments, and five periods, with 10 pots of wheat plants at each period, and each parameter was measured with 3 replications. We observed different growth stages and growth patterns of wheat under a microscope (Olympus SZ2-ILST; Tokyo, Japan) [50]. During the wheat booting stage (from spike differentiation to tetrad formation period), pots with uniformly growing plants were selected for low-temperature treatment in an artificial climate chamber (the relative humidity 70%, light intensity 0 Lx, temperature variation ±0.5 °C, temperature control range −10~40 °C, Nanjing Hengyu Instrument Equipment Manufacturing Co., Ltd., Nanjing, China). According to previous studies [26,55], the low-temperature treatment was conducted from 1:00 to 5:00 a.m. for four consecutive hours, and each pot was sprayed with 200 mL of 0.2% KH_2_PO_4_ solution after treatment, while the control pots were sprayed with an equal amount of water. The pots were then returned to their original field locations to continue growing until maturity. Other management measures were the same as the local high-yield field practices. For each treatment group of the two wheat varieties, wheat plants with consistent growth status were sampled at 0, 3, 6, 9, and 12 d DALTT (10 pots per treatment). Flag leaves from the main stems were immediately frozen in liquid nitrogen, transported back to the laboratory, and stored at −80℃ for subsequent physiological measurements. A schematic diagram outlining the experimental design and treatments is shown in Figure 8.

### 4.2. Determination of Antioxidant Enzyme Activity and Malondialdehyde Content

The leaf tissue was ground in liquid nitrogen; 2 mL 0.05 mol/L of phosphate buffer (pH = 7.8) was added and homogenized at 4 °C. The homogenate was centrifuged at 4 °C at 3000× *g* for 10 min. The supernatant was extracted for enzyme determination. The activities of superoxide dismutase (SOD, EC: 1.15.1.1), peroxidase (POD, EC: 1.11.1.7) and catalase (CAT, EC: 1.11.1.6) were determined by the nitrogen blue tetrazole colorimetric, the guaiacol colorimetric, and hydrogen peroxide methods, respectively [56].

The malondialdehyde (MDA) was determined via the thiobarbituric acid method [56]. Briefly, 0.1 g of fresh wheat leaves was weighed, and 5 mL of 5% trichloroacetic acid solution (TCA) was added. The sample was ground, and the homogenate was then centrifuged at 3000× *g* for 10 min. 2 mL of 0.67% thiobarbituric acid (TBA) was added into 2 mL of the supernatant. The mixture was boiled in a water bath at 100°C for 30 min. After cooling, it was centrifuged again, and the absorbance values of the supernatant were measured at 450 nm, 532 nm, and 600 nm using the UV-visible spectrophotometer (UV-1800, SHIMADZU, Kyoto, Japan). 

### 4.3. Determination of Osmotic Regulatory Substances

Proline (Pro) was determined by sulfosalicylic acid colorimetry [57]. The 0.1 g fresh wheat leaves were weighed, then 3 mL 3% sulfosalicylic acid solution was added, and soaked in a boiling water bath for 10 min. After the test tube was cooled to room temperature, 1.5 mL supernatant was taken, and 1.5 mL glacial acetic acid and 2.25 mL 2.5% ninhydrin solution were added, heated in a boiling water bath for 40 min, then cooled. The 3.75 mL toluene was added for full shock to extract the red substance, leaving it to be stratified and absorbing the toluene layer, and the absorption value of 520 nm was determined using the microcoder (Multiskan FC, Thermo Fisher Instruments Ltd., Waltham, MA, USA).

Soluble protein (SP) was determined by the Coomassie Brilliant Blue G-250 staining method [57]. In detail, 0.1 g of fresh wheat leaves was weighed, and 5 mL of water was added, followed by grinding to obtain a homogeneous slurry. The mixture was then centrifuged at 10,000 r/min for 10 min, and the supernatant was collected. The 4 mL of the supernatant was taken and diluted 10 times with water for later use. The 2 mL of the diluted extract was pipetted into a test tube, and 2 mL of Coomassie Brilliant Blue G-250 solution was added. The mixture was thoroughly mixed and allowed to stand for 2 min before the absorbance at 595 nm was measured. 

### 4.4. Light Microscopy

The flag leaves of the main stem of wheat with a uniform growth status at 12 DALTT were selected, and the size of 1 mm^3^ was taken in the middle of the leaves (avoiding the main vein), and fixed with 70% FAA fixation solution. The sections were put into a dewaxing transparent solution for 20 min, then soaked in 100% and 75% anhydrous ethanol (5 min each), and rinsed with clean water after treatment. The tissues were dehydrated, impregnated with wax, and encapsulated for preparation. The sections were dyed in safranin O staining solution for 3–5 s and washed with tap to remove excess dye. Next, the sections were put into 50%, 70%, and 80% alcohol for decolorization (3–8 s each). After that, the sections were put into fast green staining solution for 4–6 s and dehydrated with three cylinders of anhydrous ethanol. Finally, the sections were treated in xylene solution for 5 min, then sealed with neutral gum. The sections were scanned using a digital tissue section scanner (Pannoramic 250 FLASH, 3DHISTECH, Budapest, Hungary), and the digital section browsing software (CaseViewer 2.4, 3DHISTECH, Budapest, Hungary) was used for observation and image capture in the computer.

### 4.5. Ultrastructure Observation of Chloroplasts 

The flag leaves of the main stem of wheat with uniform growth status at 12 DALTT were harvested. Mesophyll tissues were taken from the middle of the leaves, avoiding the main vein, and immediately placed into a Petri dish with 2.5% glutaraldehyde fixing solution. The 1 mm^3^ tissue blocks were cut in the Petri dish with a scalpel. The tissue mass was transferred to a new fixation solution for fixation. The fixed sample was rinsed with 0.1 M phosphate buffer (PBS, pH = 7.4) 3 times for 15 min each time, then fixed with 1% osmic acid at room temperature and placed away from light for 7 h. The tissues were dehydrated in ethanol of different concentrations successively, then permeated and embedded. Then, the embedded plates were polymerized in an oven at 60 °C for 48 h, and the resin blocks were removed for use. The resin block was sliced on an ultra-thin microtome (LeicaUC7, Leica, Tokyo, Japan). Transmission electron microscope (HT7800, Hitachi, Tokyo, Japan) was used for observation, and 3 views were selected for image collection and analysis.

### 4.6. Determination of Photosynthetic Pigments

The contents of chlorophyll and carotenoid in the flag leaves of wheat were determined via an extraction method [58]. The 0.1 g of the fresh wheat leaves were weighed; 20 mL of 95% ethanol was added, and the leaves were soaked for 24 h until they were completely white. The absorbance of the extracts was determined by a UV-VIS spectrophotometer at 665 nm, 649 nm and 470 nm, respectively. The photosynthetic pigment content was calculated according to the following formula:

Chlorophyll a (Chla) = 13.95A_665_ − 6.88A_649_ (1)

Chlorophyll b (Chlb) =24.96A_649_ − 7.32A_665_ (2)

Carotenoid (Car) = (1000A_470_ − 2.05Chla − 114.8Chlb)/245 (3)

### 4.7. Determination of Photosynthetic Enzyme Activity

Ribulose–1,5–bisphosphate carboxylase (Rubisco, EC: 4.1.1.39) and Phosphoenolpyruvate carboxykinase (PEPC, EC: 4.1.1.32) were determined via an Boxbio assay kit (Beijing Boxbio Science and Technology Co., Ltd., Beijing, China). The 0.1 g of fresh wheat leaves were weighed, 1 mL of extraction solution was added, homogenized in an ice bath, centrifuged at 4 °C at 10,000 g/min for 10 min, and crude enzyme solution was extracted. After each reagent was added in turn according to the kit instructions, the absorbance value of 340 nm was determined by an enzyme labeling instrument.

### 4.8. Determination of Photosynthetic Parameters

At 0, 3, 6, 9, and 12 DALTT, flag leaves with basically the same growth status were used for each treatment. The net photosynthetic rate (Pn), stomatal conductivity (Gs), transpiration rate (Tr), and intercellular CO_2_ concentration (Ci) of the wheat leaves were determined via a portable photosynthetic rate analyzer LI-6800 (LI-COR, Lincoln, NE, USA). The photosynthetically active radiation (PAR) was set at 1200 μmol m^−2^ s^−1^, the leaf chamber temperature was controlled at 25 °C, and the carbon dioxide (CO_2_) concentration was controlled at about 400 μmol·mol^−1^. Three leaves were randomly chosen and measured between 9:00 and 11:00 a.m. for each treatment.

### 4.9. Yield and its Component Factors

At maturity, 10 plants with a uniform growth status were selected from each treatment of two wheat varieties and brought indoors for yield analysis. Yield components such as the grain number per spike, the 1000-grain weight, and the grain yield per stem were measured. Additionally, the yield loss rate and yield recovery rate were calculated.

The formula for calculating the yield loss rate is:

(4) $$R_{YL}=\frac{Y_{T}-Y_{CK}}{Y_{CK}}$$

The formula for calculating the yield recovery rate is:

(5) $$R_{YR}=\frac{Y_{T2}-Y_{T1}}{Y_{CK}-Y_{T1}}$$

In the formula, R_YL_ represents the yield loss rate (%), and R_YR_ represents the yield recovery rate (%). Y_T_ refers to the grain yield per stem (g) via LT1 or LT2, and Y_CK_ refers to the grain yield per stem (g) in CK. Y_T1_ represents the grain yield per stem (g) via LT1, and Y_T2_ represents the grain yield per stem (g) via LT2 treatment.

### 4.10. Statistical Analyses

Excel 2021 (Microsoft, Washington, DC, USA), SPSS 27 (IBM Corp., Armonk, Chicago, IL, USA), and the "corrplot" package in RStudio were used for basic data statistics, data processing, and correlation analysis, respectively. Image-J analysis software (National Institutes of Health, Bethesda, MD, USA) was used to measure leaf thickness, upper epidermis thickness, lower epidermis thickness, palisade tissue thickness, spongy tissue thickness, chloroplast area, chloroplast length and width, and granule thickness. The results were expressed as means ± SD. Duncan’s method was used to determine significant differences at the *p <* 0.05 level. The correlation between the traits was analyzed using Pearson’s correlation coefficient (r).

## 5. Conclusions

The present study indicated that spring LTS increased the activities of the SOD, the POD, the CAT and the MDA content, and damaged the leaf cell morphology and tissue integrity, resulting in decreased photosynthetic pigment content and photosynthetic enzyme activities, ultimately depressing the photosynthetic functions of wheat flag leaves and reducing the wheat yield. Spraying KH_2_PO_4_ can increase photosynthetic pigment content, photosynthetic enzyme activities, and Pn in wheat flag leaves by enhancing the activities of SOD, POD, and CAT, and decreasing the MDA content and increasing the accumulation of Pro and SP, finally alleviating the damage to leaf cell caused by spring LTS, leading to improved wheat grain yield. Therefore, spraying KH_2_PO_4_ is meaningful and practical for improving wheat resistance to spring LTS, especially combined with modern unmanned drone flight defense, which has great application prospects in wheat production.

## Figures and Tables

**Table 1:** Comparison of antioxidant activities and MDA contents in flag leaves of wheat varieties Yannong19 (YN19) and Xinmai26 (XM26) in different treatments.

Variety	Days (d)	Treatment	SOD (U g^−1^ FW)	POD (U g^−1^ FW)	CAT (U g^−1^ FW)	MDA (μmol g^−1^ FW)
YN19	0	CK	409.36 ± 10.05 b	26.38 ± 2.66 b	371.53 ± 12.23 b	15.43 ± 1.09 b
		LT1	688.12 ± 6.59 a	62.14 ± 2.30 a	447.99 ± 4.29 a	23.95 ± 0.88 a
		LT2	688.12 ± 6.59 a	62.14 ± 2.30 a	447.99 ± 4.29 a	23.95 ± 0.88 a
	3	CK	428.46 ± 6.37 b	33.04 ± 2.38 b	377.40 ± 4.49 b	15.90 ± 0.73 b
		LT1	696.87 ± 4.48 a	64.38 ± 3.52 a	453.89 ± 9.73 a	25.21 ± 0.43 a
		LT2	707.64 ± 6.38 a	68.89 ± 4.03 a	462.29 ± 4.17 a	24.46 ± 1.21 a
	6	CK	449.97 ± 12.54 c	38.62 ± 3.22 c	389.45 ± 4.80 b	16.69 ± 1.34 c
		LT1	707.63 ± 6.86 b	69.78 ± 2.61 b	458.56 ± 3.54 a	29.95 ± 0.20 a
		LT2	738.35 ± 21.85 a	77.34 ± 1.60 a	469.92 ± 13.13 a	25.81 ± 2.37 b
	9	CK	467.18 ± 3.32 c	45.49 ± 0.93 c	394.42 ± 3.23 c	18.81 ± 0.62 c
		LT1	657.31 ± 11.66 b	63.60 ± 5.23 b	431.83 ± 4.07 b	32.05 ± 1.45 a
		LT2	709.94 ± 4.15 a	75.41 ± 2.94 a	464.77 ± 7.06 a	26.84 ± 1.77 c
	12	CK	485.24 ± 6.99 c	53.36 ± 3.62 c	383.52 ± 2.05 c	19.70 ± 1.75 c
		LT1	606.25 ± 7.09 b	60.67 ± 0.86 b	422.50 ± 8.76 b	36.44 ± 2.05 a
		LT2	688.75 ± 4.28 a	71.78 ± 0.28 a	455.01 ± 7.12 a	29.27 ± 1.23 b
XM26	0	CK	381.17 ± 3.85 b	23.38 ± 1.79 b	342.65 ± 11.64 b	17.22 ± 1.12 b
		LT1	621.63 ± 27.92 a	42.61 ± 2.08 a	406.79 ± 4.08 a	29.02 ± 1.00 a
		LT2	621.63 ± 27.92 a	42.61 ± 2.08 a	406.79 ± 4.08 a	29.02 ± 1.00 a
	3	CK	403.19 ± 2.20 c	24.04 ± 1.48 b	354.79 ± 2.79 b	18.03 ± 1.43 b
		LT1	647.89 ± 9.95 b	53.27 ± 3.80 a	444.88 ± 8.44 a	30.15 ± 0.85 a
		LT2	666.77 ± 5.80 a	55.96 ± 3.91 a	452.60 ± 3.90 a	30.01 ± 1.17 a
	6	CK	425.74 ± 4.09 c	34.45 ± 2.50 c	378.90 ± 13.43 c	19.37 ± 0.89 c
		LT1	655.41 ± 5.07 b	55.38 ± 2.13 b	461.82 ± 9.74 b	35.56 ± 1.20 a
		LT2	686.45 ± 6.53 a	60.33 ± 0.86 a	481.57 ± 3.45 a	32.01 ± 0.66 b
	9	CK	439.56 ± 6.94 c	37.14 ± 2.77 c	374.47 ± 10.30 c	21.34 ± 1.80 c
		LT1	618.28 ± 8.20 b	52.68 ± 0.91 b	449.74 ± 1.25 b	39.30 ± 1.41 a
		LT2	663.57 ± 8.28 a	61.90 ± 0.04 a	477.37 ± 3.54 a	32.24 ± 0.93 b
	12	CK	456.27 ± 2.08 c	42.72 ± 1.82 c	368.43 ± 3.31 c	22.69 ± 1.03 c
		LT1	577.70 ± 2.79 b	48.73 ± 0.45 b	422.12 ± 3.69 b	42.47 ± 2.10 a
		LT2	646.29 ± 9.79 a	55.90 ± 2.35 a	464.57 ± 1.42 a	35.24 ± 0.78 b

The standard deviation (SD) value from three replicates is represented as error bars, and different lowercase letters indicate significant differences at the *p <* 0.05 level. Legend: SOD—superoxide dismutase; POD—peroxidase; CAT—catalase; MDA—malondialdehyde. CK refers to treatment at 15 °C and spraying H_2_O; LT1 refers to treatment at −4 °C and spraying H_2_O; LT2 refers to treatment at −4 °C and spraying KH_2_PO_4_.

**Table 2:** Comparison of proline and soluble protein contents in flag leaves of wheat varieties Yannong19 (YN19) and Xinmai26 (XM26) in different treatments.

Variety	Days (d)	Treatment	Proline (μg g^−1^ FW)	Soluble Protein (mg g^−1^ FW)
YN19	0	CK	128.45 ± 6.63 b	15.52 ± 0.08 b
		LT1	143.90 ± 4.21 a	26.06 ± 1.55 a
		LT2	143.90 ± 4.21 a	26.06 ± 1.55 a
	3	CK	129.78 ± 6.46 b	17.15 ± 1.22 b
		LT1	144.59 ± 6.74 a	28.30 ± 0.32 a
		LT2	146.61 ± 4.81 a	29.00 ± 1.62 a
	6	CK	141.36 ± 4.44 b	19.11 ± 1.18 c
		LT1	147.37 ± 2.19 ab	30.19 ± 1.73 b
		LT2	154.82 ± 4.72 a	36.23 ± 1.88 a
	9	CK	144.45 ± 3.84 c	24.11 ± 1.73 c
		LT1	155.49 ± 1.91 b	31.60 ± 2.35 b
		LT2	169.02 ± 0.48 a	37.74 ± 2.67 a
	12	CK	150.83 ± 4.93 c	27.46 ± 2.08 c
		LT1	166.48 ± 6.67 b	34.00 ± 3.98 b
		LT2	188.39 ± 5.32 a	41.21 ± 2.15 a
XM26	0	CK	127.21 ± 3.58 b	11.83 ± 0.49 b
		LT1	142.17 ± 0.37 a	19.52 ± 2.44 a
		LT2	142.17 ± 0.37 a	19.52 ± 2.44 a
	3	CK	128.19 ± 4.24 b	14.57 ± 2.62 b
		LT1	152.30 ± 4.37 a	22.42 ± 0.81 a
		LT2	154.31 ± 2.54 a	23.01 ± 0.26 a
	6	CK	139.59 ± 5.68 b	17.69 ± 2.01 b
		LT1	153.69 ± 5.39 a	25.72 ± 2.01 a
		LT2	156.62 ± 0.98 a	28.43 ± 3.00 a
	9	CK	141.69 ± 7.60 b	19.33 ± 1.46 c
		LT1	158.22 ± 4.56 a	25.49 ± 0.96 b
		LT2	166.51 ± 1.24 a	32.74 ± 1.92 a
	12	CK	142.57 ± 4.35 c	22.86 ± 3.35 c
		LT1	160.67 ± 7.70 b	30.62 ± 0.45 b
		LT2	174.72 ± 3.60 a	36.59 ± 1.79 a

The standard deviation (SD) value from three replicates is represented as error bars, and different lowercase letters indicate significant differences at the *p <* 0.05 level. CK refers to treatment at 15 °C and spraying H_2_O; LT1 refers to treatment at −4 °C and spraying H_2_O; LT2 refers to treatment at −4 °C and spraying KH_2_PO_4_.

**Table 3:** Comparison of leaf tissue thickness of flag leaf between Yannong19 (YN19) and Xinmai26 (XM26) in different treatments.

Variety	Treatment	Leaf Thickness (μm)	UE Thickness (μm)	LE Thickness (μm)	PT Thickness (μm)	ST Thickness (μm)	Tightness of the Blade Structure	Blade Structure Dredging Density
YN19	CK	190.63 ± 0.35 a	15.23 ± 0.06 a	14.50 ± 0.30 a	55.53 ± 0.21 a	44.63 ± 0.21 a	0.29 ± 0.0006 a	0.23 ± 0.0009 a
	LT1	156.30 ± 2.51 c	7.10 ± 0.10 c	8.06 ± 0.15 c	30.00 ± 0.44 c	25.67 ± 0.25 c	0.19 ± 0.0055 c	0.16 ± 0.0022 c
	LT2	172.63 ± 1.55 b	10.60 ± 0.10 b	11.37 ± 0.25 b	40.70 ± 0.92 b	33.57 ± 0.15 b	0.24 ± 0.0033 b	0.19 ± 0.0017 b
XM26	CK	179.00 ± 1.35 a	13.73 ± 0.06 a	12.60 ± 0.10 a	48.90 ± 0.17 a	37.40 ± 0.20 a	0.27 ± 0.0029 a	0.21 ± 0.0015 a
	LT1	146.53 ± 1.86 c	6.60 ± 0.10 c	7.53 ± 0.15 c	26.07 ± 0.42 c	23.73 ± 0.15 c	0.18 ± 0.0011 c	0.16 ± 0.0031 c
	LT2	164.03 ± 1.44 b	9.70 ± 0.10 b	10.60 ± 0.20 b	35.27 ± 0.42 b	30.30 ± 0.46 b	0.22 ± 0.0033 b	0.18 ± 0.0012 b

Tightness of the blade structure (%) = (Palisade tissue thickness/Leaf thickness) × 100%. Blade structure dredging density (%) = (Sponge tissue thickness/Leaf thickness) × 100%. Fence tissue sponge tissue ratio = Palisade tissue thickness/Sponge tissue thickness. Data are means ± standard deviation. Different lowercase letters indicate significant differences at the *p <* 0.05 level. Legend: UE—upper epidermis; LE—lower epidermis; PT—palisade tissue; ST—spongy tissue. CK refers to treatment at 15 °C and spraying H_2_O; LT1 refers to treatment at −4 °C and spraying H_2_O; LT2 refers to treatment at −4 °C and spraying KH_2_PO_4_.

**Table 4:** Comparison of chloroplast structure parameters of flag leaves between Yannong19 (YN19) and Xinmai26 (XM26) under different treatments.

Variety	Treatment	Number of Chloroplasts	Chloroplast Area (μm^2^)	Chloroplast Length/Width
YN19	CK	19.67 ± 2.08 a	24.67 ± 1.53 a	2.42 ± 0.02 a
	LT1	7.00 ± 1.00 c	11.67 ± 1.53 c	1.65 ± 0.02 c
	LT2	13.67 ± 1.53 b	16.33 ± 1.15 b	1.91 ± 0.02 b
XM26	CK	17.00 ± 1.73 a	21.33 ± 1.53 a	2.27 ± 0.02 a
	LT1	6.33 ± 0.58 c	9.67 ± 0.58 c	1.51 ± 0.02 c
	LT2	12.33 ± 1.15 b	14.00 ± 1.00 b	1.84 ± 0.02 b

Data are mean ± standard deviation. Different lowercase letters indicate significant differences at the *p <* 0.05 level. CK refers to treatment at 15 °C and spraying H_2_O; LT1 refers to treatment at −4 °C and spraying H_2_O; LT2 refers to treatment at −4 °C and spraying KH_2_PO_4_.

**Table 5:** Comparison of flag leaf thylakoid system parameters between Yannong19 (YN19) and Xinmai26 (XM26) in different treatments.

Variety	Treatment	Number of Grana	Number of Grana Lamellae	Grana Thickness (μm)
YN19	CK	21.67 ± 0.58 a	20.67 ± 0.58 a	0.25 ± 0.02 a
	LT1	9.33 ± 1.53 c	8.33 ± 1.53 c	0.14 ± 0.02 c
	LT2	15.33 ± 1.53 b	15.67 ± 1.53 b	0.19 ± 0.02 b
XM26	CK	19.33 ± 1.53 a	18.67 ± 1.53 a	0.21 ± 0.02 a
	LT1	6.00 ± 1.00 c	6.00 ± 1.00 c	0.13 ± 0.02 c
	LT2	12.67 ± 1.53 b	14.33 ± 1.53 b	0.17 ± 0.02 b

Data are mean ± standard deviation. Different lowercase letters indicate significant differences at the *p <* 0.05 level. CK refers to treatment at 15 °C and spraying H_2_O; LT1 refers to treatment at −4 °C and spraying H_2_O; LT2 refers to treatment at −4 °C and spraying KH_2_PO_4_.

**Table 6:** Comparison of yield and its component factors between Yannong19 (YN19) and Xinmai26 (XM26) in different treatments.

Variety	Treatment	GNPS	TGW (g)	GYPS (g)	YLR (%)	YRR (%)
YN19	CK	44.33 ± 2.08 a	47.49 ± 0.48 a	2.68 ± 0.04 a	-	-
	LT1	29.33 ± 1.53 c	35.64 ± 0.46 c	1.70 ± 0.02 c	36.57	-
	LT2	36.33 ± 2.31 b	42.91 ± 0.96 b	2.05 ± 0.03 b	23.51	35.71
XM26	CK	48.00 ± 1.00 a	46.21 ± 1.89 a	2.75 ± 0.02 a	-	-
	LT1	19.00 ± 0.00 c	32.39 ± 0.52 c	1.31 ± 0.06 c	52.36	-
	LT2	27.00 ± 0.00 b	38.10 ± 0.53 b	1.77 ± 0.04 b	35.64	31.94

Data are means ± standard deviation. Different lowercase letters indicate significant differences at the *p <* 0.05 level. Legend: GNPS—grain number per spike; TGW—1000-grain weight; GYPS—grain yield per stem; YLR—yield loss rate; YRR—yield recovery rate. CK refers to treatment at 15 °C and spraying H_2_O; LT1 refers to treatment at −4 °C and spraying H_2_O; LT2 refers to treatment at −4 °C and spraying KH_2_PO_4_.

## Data Availability

Data are contained within the article.
